# Implementation fidelity of a self-management course for epilepsy: method and assessment

**DOI:** 10.1186/s12874-017-0373-x

**Published:** 2017-07-11

**Authors:** G. Wojewodka, S. Hurley, S. J. C. Taylor, A. J. Noble, L. Ridsdale, L. H. Goldstein

**Affiliations:** 1 King’s College London, Institute of Psychiatry, Psychology and Neuroscience, De Crespigny Park, London, SE5 8AF UK; 2 Centre for Primary Care and Public Health, Bart’s and the London School of Medicine and Dentistry, Queen Mary University of London, London, UK; 3 Institute of Psychology, Health and Society, University of Liverpool, Liverpool, UK; 4 King’s College London, Institute of Psychiatry, Psychology and Neuroscience, PO 77, The Henry Wellcome Building, De Crespigny Park, London, SE5 8AF UK

**Keywords:** Fidelity, Complex intervention, Adherence, Competence, Didacticism, Epilepsy, Self-management

## Abstract

**Background:**

Complex interventions such as self-management courses are difficult to evaluate due to the many interacting components. The way complex interventions are delivered can influence the effect they have for patients, and can impact the interpretation of outcomes of clinical trials. Implementation fidelity evaluates whether complex interventions are delivered according to protocol. Such assessments have been used for one-to-one psychological interventions; however, the science is still developing for group interventions.

**Methods:**

We developed and tested an instrument to measure implementation fidelity of a two-day self-management course for people with epilepsy, SMILE(UK). Using audio recordings, we looked at adherence and competence of course facilitators. Adherence was assessed by checklists. Competence was measured by scoring group interaction, an overall impression score and facilitator “didacticism”. To measure “didacticism”, we developed a novel way to calculate facilitator speech using computer software. Using this new instrument, implementation fidelity of SMILE(UK) was assessed on three modules of the course, for 28% of all courses delivered.

**Results:**

Using the instrument for adherence, scores from two independent raters showed substantial agreement with weighted Kappa of 0.67 and high percent agreement of 81.2%. For didacticism, the results from both raters were highly correlated with an intraclass coefficient of 0.97 (*p* < 0.0001). We found that the courses were delivered with a good level of adherence (> 50% of scored items received the maximum of 2 points) and high competence. Groups were interactive (mean score: 1.9–2.0 out of 2) and the overall impression was on average assessed as “good”. Didacticism varied from 42% to 93% of total module time and was not associated with the other competence scores.

**Conclusion:**

The instrument devised to measure implementation fidelity was reproducible and easy to use. The courses for the SMILE(UK) study were delivered with a good level of adherence to protocol while not compromising facilitator competence.

**Trial registration:**

ISRCTN57937389.

**Electronic supplementary material:**

The online version of this article (doi:10.1186/s12874-017-0373-x) contains supplementary material, which is available to authorized users.

## Background

Evaluating the effectiveness of complex interventions, such as a group self-management course, can be challenging. They include multiple, often interacting components and thus present a host of problems for researchers trying to evaluate their impact upon patient outcomes. Although such interventions typically follow specific therapeutic models, protocols and manuals, one important task is to document the extent and way in which the intervention was actually implemented, not least because this can be a mediator of study outcomes [1]. This issue is often neglected [2]. Despite this, measuring a complex intervention’s implementation fidelity, that is the degree to which the treatment/programme was delivered as intended, is a relatively recent academic endeavour. The Medical Research Council (MRC) in the United Kingdom (UK) devised an evaluation framework in order to help researchers develop and evaluate complex interventions effectively [3]. It stressed the importance of evaluating fidelity. However, *how* to measure fidelity is still a developing science. The National Institute of Health Behaviour Change Consortium from the United States has presented five aspects of fidelity: study design, training of intervention providers, delivery of intervention, receipt of intervention and enactment of skills [1]. The study design must allow for accurate replication of the intervention. Those delivering the intervention, here (in the context of SMILE(UK) referred to as facilitators), should be provided with adequate training to know how to deliver the intervention as intended. Next, the way participants receive the intervention and implement what they have learned can vary, and thus affect the study. Fidelity assessments can look at all these five aspects [4]. In the study presented here, we focus on delivery of the intervention or how it was implemented. Information about the study design [5, 6] and enactment of skills as determined by a process evaluation [7] can be found elsewhere.

Although definitions of implementation fidelity vary greatly across studies, work has begun to measure fidelity via the constructs of “adherence” and “competence” [8]. Adherence is defined as the extent to which the core content of a programme was delivered as instructed, including specific topics and techniques to use and those to avoid [9, 10]. High adherence requires rigidity to instructions and knowledge of how to deliver each component as required by the protocol. Competence, in contrast, focuses on the quality with which the facilitators delivered the intervention, or “how” the information should be provided. It takes into account, where relevant, factors such as appropriate pacing, communication skills and the manner of interaction with the intervention’s recipient(s) [9]. It relies on the expertise and judgement of facilitators to deliver specific topics while adapting to ensure the intervention meets the group’s needs, thus requiring flexibility [11]. Since the assessment of facilitator competence is a relatively new area of investigation, it is less likely to be reported in studies that measure fidelity [9]. Arguably, this is an oversight, since a person may deliver an intervention’s content as prescribed, but do it in a way that is poorly timed, badly communicated, or which neglects variability in the needs and learning abilities of the recipient/s [12]. Low competence may affect intervention receipt and subsequently enactment of skills [11]. Adherence and competence, or rigidity versus flexibility, should be balanced to consistently deliver a complex intervention suitable for a specific context [3, 11].

To date, most efforts within the literature have focused on measuring the fidelity of one-to-one psychotherapy-style interventions [8, 13]. Complex interventions are, however, frequently delivered within group formats. It cannot be assumed that techniques and approaches developed for individual psychological treatments will easily extend to the group format. For example, one aspect of competence that is largely neglected within evaluations of individual treatments but which is of potential importance within group interventions is recipient-facilitator interactivity, or in other words, the degree of “didacticism” displayed by the facilitator. One proxy for didacticism is the proportion of time the facilitator (rather than the recipients) spends speaking during course delivery. A highly didactic intervention is likely to have a facilitator speaking for large amounts of time without engaging with the recipient(s), and has been shown to be less effective in producing behaviour change [14]. A certain level of didacticism is, however, needed so participants remain oriented to the goals of the intervention, topics introduced for discussion and certain information provided [15, 16]. Interventions that provide a mix of didactic and interactive components achieve the best outcomes for behaviour change [14].

There have been a number of studies testing group self-management courses for people with epilepsy; however, to date and to our knowledge, none has evaluated implementation fidelity [17–28]. In the context of a randomised controlled trial, we looked to assess implementation fidelity of a two-day group self-management course for epilepsy (Self-Management course for people with poorly controlled epILEpsy, SMILE(UK)) [5]. We aimed to do this by: 1) developing an instrument incorporating measures of both adherence and competence, including considerations of didacticism, and 2) using this instrument to evaluate implementation fidelity of SMILE(UK).

## Methods

### Study setting

The implementation fidelity assessment was part of a multi-centre, pragmatic, parallel group randomised controlled trial. For the trial, participants were recruited from epilepsy clinics around London and South East England. Inclusion criteria were: being 16 or older, having a documented diagnosis of epilepsy, having two seizures in the previous 12 months and being prescribed antiepileptic drugs. Exclusion criteria were: only having seizures not related to epilepsy (psychogenic or due to substance misuse) and not being able to participate in a two-day course in English and/or complete questionnaires. Participants were randomised 1:1 into the intervention group or treatment-as-usual control group. The study enrolled 404 participants, with ages ranging from 16 to 85 years old, and a median 18 years since epilepsy diagnosis. The group was highly educated, with 53% having post-secondary school qualifications and half were in employment. The trial’s primary outcome was quality of life one year after randomisation. Other measures included seizure frequency, psychological distress, felt-stigma, self-mastery, medication adherence and health economics [5, 6].

### The intervention

SMILE(UK) is a two-day self-management intervention for people with epilepsy in improving quality of life, in comparison to treatment as usual [5]. The intervention and accompanying materials were adapted for the UK population from the German course known as Modular Service Package Epilepsy (MOSES) [19]. MOSES was originally devised with the aim of improving patients’ knowledge about epilepsy, so that they can better understand its consequences and how it is diagnosed and treated, and had the aim of increasing patients’ comprehension of work-related and psychosocial problems, to enable them to become “experts” in managing their condition.

Materials used at the SMILE(UK) courses included a facilitator’s manual, participant’s workbook and a slide show. The delivery of the intervention follows the facilitator’s manual in terms of the content to be covered and outlines the teaching techniques to be used at different stages. The facilitators’ manual corresponds to the content in the participants’ workbooks. The course was designed to be interactive and a range of educational techniques (e.g. mind maps, flipchart exercises and slides) were used to promote discussion. In a similar manner to the original MOSES package, the SMILE(UK) intervention contains nine modules: Living with Epilepsy, People with Epilepsy, Basic Knowledge, Diagnosis, Treatment, Self-control, Prognosis, Personal and Social Life, and Network Epilepsy [5]. Twelve course facilitators were trained during a two-day training session led by MOSES experts. Each course was then delivered by two facilitators: an Epilepsy Nurse Specialist and a Clinical Physiologist who worked in varying pairings across the study. The intended group size was 8–12 participants; however in practice, group sizes varied more. Carers were also included if participants required help with travel and assistance during the course days. The course was piloted with volunteers prior to beginning the trial [29].

The study was approved by the National Research Ethics Service Committee London – Fulham (reference [12]/LO/1962). Trial registration: ISRCTN57937389. All participants were adults and they, themselves, gave written informed consent to enrol in the study.

### Training of facilitators

Facilitators were selected by interview by two investigators (LR and AJN). They were recruited from clinics around London and South East England. Facilitators attended a two-day training session led by experts from the MOSES course from Germany. Different techniques were described and flexibility of course material was discussed. The importance of engaging participants with epilepsy (and their carers) in sessions to make the course interactive was stressed. Indeed, as one of the goals of the intervention is that recipients talk during the course about their own experiences so they can learn from each other, those delivering the course were considered to be discussion facilitators, rather than trainers or teachers. During the training, the facilitators gained practice in the different methods to use and watched videos. Subsequent support was available in the form of mentoring sessions provided by Dr. Franz Brunnhuber, the Lead (Consultant) Neurophysiologist at King’s College Hospital (London, UK), who had himself previously trained as a MOSES facilitator. In addition, the two-day training course was video-recorded and available to facilitators to act as a reminder of the required course delivery activities. The recording was also available to new facilitators who joined the project later on due to staff turnover in the various services involved, and who were required to have observed a SMILE(UK) course before delivering one.

### Courses

Eighteen two-day courses were delivered by a total of 12 different facilitators, 11 trained facilitators (Epilepsy Nurse Specialists and Clinical Physiologists) and the study’s chief investigator (LR) who had also attended and participated in the two-day training course. The intervention arm of the trial had 205 participants, of whom 126 participants completed the full two days of the course. Course size was between six and 13 participants.

### Components evaluated

All courses were audio-recorded using a digital dictaphone (Olympus voice recorder DS-2500) and recordings were stored on a secure network. These formed the basis of the evaluations of implementation fidelity and facilitators were aware of this. Based on methods from previous studies recommending that at least 25% of recordings should be evaluated [30], the recordings from 28% of the courses (i.e. from five courses) were selected for implementation fidelity analysis once all the courses had been delivered. They were purposively selected by a study researcher (GW) who did not listen to any of the audio recordings, to ensure that courses were not chosen according to content. The different facilitator team combinations were assessed to select five courses in a way that each facilitator would be evaluated once, thus optimising variability across the sample. Since two facilitators delivered each course, the maximum number of facilitators of five courses that could be evaluated was ten. A second factor in course selection was the quality of audio recordings which was assessed by a second researcher (SH). The final selection of courses satisfied both factors. The five courses selected for evaluation of implementation fidelity were courses number 1, 8, 10, 13, and 15. These courses were delivered by 10 out of 12 facilitators. The number of participants attending the five rated courses ranged from six to 13.

### Developing the intervention fidelity measurement instrument

The development of the instrument to measure intervention fidelity was based on previous approaches where a proportion of total components was evaluated [10, 12]. A multidisciplinary team with expertise in epilepsy, psychology and self-management education first identified what they considered to be the core modules of the SMILE(UK) course (i.e. those deemed to most likely to drive behaviour change). Of the nine modules, the first eight were identified. The number of modules whose delivery was to be rated was then further reduced after referring to qualitative interviews conducted with participants attending pilot SMILE(UK) courses [29]. The volunteer participants identified *Module 3: Basic Knowledge*, *Module 4: Diagnosis* and *Module 6: Self-Control* as particularly useful. A fourth module, *Module 8: Personal and Social Life*, was also identified as helpful, although due to the variable content covered by this module, which is guided predominantly by participant choice as to input provided, it was excluded from fidelity assessment.

The three SMILE(UK) modules identified for fidelity ratings focus primarily on participant education, via the provision of information on and discussion about: the clinical science underpinning diagnosis, different treatment options, and personal assessment relating to seizures (e.g. describing seizure types, identifying triggers and warnings). They are the most factual modules of the course with specific items for facilitators to deliver; as noted above the delivery of factual content for Module 8 will vary according to participants’ choice of topics to cover so is less suitable for fidelity evaluation using the present methods. The remaining SMILE(UK) modules depend more on participants sharing personal experiences and the facilitators’ role is more limited, with techniques and questions being specified by the manual for them to use to promote group discussions The aim of those modules is more about participants becoming comfortable in sharing stories, and gaining confidence, rather than education.

#### Adherence

To measure adherence to the intended intervention content, a checklist was created for each module listing the core items that needed to be delivered according to the facilitators’ manual. Items were scored from 0 to 2 (0 = item not delivered, 1 = item partially delivered, 2 = item fully delivered). There were six adherence items each for Modules 3 and 4 (with a maximum score of 12 per module) and five adherence items for Module 6 (maximum score of 10) [see Additional file 1 for checklist].

#### Competence

Facilitator competence was evaluated using four measures: group interaction, an overall impression score, “didacticism” and facilitator techniques.

To evaluate group interaction, an extra item was added to every module checklist: “Did facilitators engage participant involvement?” [see Additional file 1]. In order to make the item rating as objective as possible, a score of 0 indicated that one course participant had dominated discussions, 1 indicated that two-to-three participants had interacted with facilitators, and 2 indicated that four or more participants had interacted in the session.

Similar to other studies [10], we included an “overall impression” measure to gauge how well each module was delivered [see Additional file 1]. Raters used a scale of 1 to 4. A score of 1 was defined as “Poor: largely didactic session, little participant input, little group cohesion”. A rating of 2 was defined as “Average: mostly didactic session, some participant input, some group discussion”. A rating of 3 was defined as “Good: some didactic teaching, significant participant input and group discussion”. A rating of 4 was defined as “Excellent: minimal didactic teaching, substantial group input and discussion”.

Using the annotation software ELAN (Max Planck Institute for Psycholinguistics, The Language Archive, Nijmegen, The Netherlands, http://tla.mpi.nl/tools/tla-tools/elan/) [31] raters were able to record objectively the total amount of facilitator speech (expressed in seconds) as a proxy measure of how “didactic” the delivery of each module was. Every instance of facilitator speech was recorded. However, any filler words delivered by facilitators (e.g., “oh”; “okay”; “yeah”) were defined as non-instances of facilitator speech. Once the total time of facilitator speech was determined, it was then divided by the duration for the delivery of the module. The result was expressed as a percentage of total module delivery time.

Each module checklist also enabled the type and number of times facilitator techniques that were used (e.g. flipchart exercises, slides) to be recorded throughout the delivery of each module [see Additional file 1].

#### Testing the instrument

The fidelity instrument was first piloted by two members of the research team to check its feasibility. Then, in order to minimise bias, two raters who were independent from the SMILE(UK) research team were recruited to complete the measurements. Raters first received training by conducting pilot ratings on SMILE(UK) courses delivered to control participants (i.e., participants allocated to the standard medical care arm of the trial who received a course at the end of the trial). To clarify any misunderstanding of how items should be rated, a scoring guide was developed which included a list of specific topics that would need to be delivered to receive a full score. For example, to receive a full score for “seizure types”, the following topics would have to be addressed: generalised tonic-clonic, absence, complex partial, simple partial and myoclonic.

### Data analysis

Descriptive statistics are shown as means and ranges. Inter-rater reliability was measured by using the weighted Kappa statistic with linear weighting for ordinal values (checklist scores) and by the intraclass coefficient for continuous values (didacticism measurements). Percent agreement was also calculated for checklist ratings to determine how often the same scores were given by both raters. Adherence scores given by both raters were averaged and the frequency of each score was illustrated as a percentage of total items per module. Competence scores were averaged and tabulated for each module. We used simple regression analysis to look at associations between different categories of ratings, looking at didacticism (independent variable) as a potential predictor of adherence, and overall impression. The associations between length of module (independent variable) and overall impression were also tested, as was group size (independent variable) as a predictor of didacticism. The associations between didacticism and adherence and overall impression, and length of module and overall impression were also tested using group size as a covariate. Results are reported in terms of beta coefficients (β) and 95% confidence intervals.

## Results

### Evaluating the fidelity instrument

#### Inter-rater reliability: adherence

Ratings were performed on 15 distinct sessions (three modules across five courses) and a total of 85 items were scored (Table 1). The results from the two independent raters were tabulated and assessed for inter-rater reliability of the adherence checklist. There was a substantial agreement between the ratings with a weighted Kappa of 0.67 [32]. Percent agreement was high (81.2%).

**Table 1:** SMILE (UK) components evaluated

Components evaluated	Description of module	Number of adherence items rated	Number of competence items rated
Module 3: Basic Knowledge	What causes seizures; how do seizures develop in the brain; what are different seizure types	6	3
Module 4: Diagnosis	What information is needed about seizures to help with diagnosis (observing, describing, documenting seizures); how to understand different methods for diagnosis	6	3
Module 6: Self-Control	What can trigger seizures and how to avoid them; what is an aura (i.e. signs occurring before seizures); developing abilities for self-control	5	3

#### Inter-rater reliability: competence

A similar analysis was undertaken on the overall impression component of the competence measure. Although this was a subjective measure, there was substantial agreement between the two raters with a Kappa of 0.65 [32]. The percent agreement was 60.0%.

To evaluate the novel measure of didacticism, the intraclass correlation coefficient was calculated for this measure of competence. The results from both raters were highly correlated with a coefficient of 0.97 (*p* < 0.0001), thus demonstrating the high reproducibility of this method.

As the scores for participant involvement were almost all the maximum of 2, inter-rater agreement was not calculated for this measure.

### Evaluating course fidelity

#### Adherence results

**Fig. 1:**
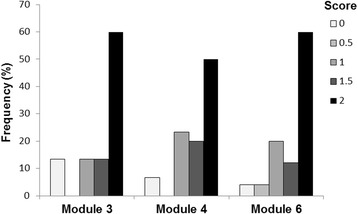
Frequency of scores for adherence items. Adherence scores from both raters were averaged and the frequencies of each score were calculated as a percentage of all items for that module. Modules 3 and 4 had each 30 items, and Module 6 had 25 items scored by two raters

The three selected modules were evaluated for five SMILE(UK) courses (Table 1). Scores from both raters were averaged for each module and the frequencies of scores are presented in Fig. 1. The maximum score of 2 was given to the majority (50–60%) of all adherence items. Module 3 had the most non-delivered items.

We then looked at the scores for individual items to assess how specific components were delivered [see Additional file 2]. Four items were fully delivered across all five courses: how seizures develop, seizure types, noticing events pre/during/post-seizures, and seizure triggers. In the whole adherence assessment, a score of 0 (item not delivered) was given seven times: five times for items relating to completing the participant workbook, one item regarding seizures and one item about auras (i.e. warning signs prior to seizures) [Additional file 2, Table 1].

#### Competence results

The evaluation of group interaction was based on the number of participants judged to have spoken during a module (Table 2). All but two sessions were given a full score of 2, indicating a high interactivity with four or more participants speaking during the sessions.

**Table 2:** Intervention fidelity: competence ratings

Component	Group interaction mean score (range)	Overall impression mean score (range)	Didacticism mean % (range)
Module 3: Basic Knowledge	1.9 (1.5–2.0)	2.6 (1.5–3.5)	71 (55–78)
Module 4: Diagnosis	1.9 (1.5–2.0)	2.6 (1.0–4.0)	69 (48–93)
Module 6: Self-Control	2.0 (2.0–2.0)	3.0 (2.0–4.0)	58 (42–76)

Overall impression was assessed using a scale of 1 to 4. Twelve sessions had average-to-excellent delivery and only three sessions were given scores below 2, indicating poor delivery. The maximum score was attained for some Module 4 and 6 sessions (Table 2).

The analysis of “didacticism” revealed a broad range of results, from 42% to 93% of facilitator speech during the sessions. The greatest range of didacticism was seen for the delivery of Module 4 (Table 2). Using simple regression analysis, we found no associations between didacticism and adherence (β = 0.23 [95% CI: -0.27 – 0.87]), or between didacticism and overall impression scores (β = 0.081 [95% CI: -0.52 – 0.68]). Additionally, we looked at whether the length of the session was related to overall impression scores and found no association between the two (β = 0.13 [95% CI: -0.46 – 0.73]). Adding group size as a covariate did not change the results of the regression analyses. Didacticism was predicted by group size, whereby smaller groups were associated with less didactic sessions (β = 0.60 [95% CI: 0.12–1.08]).

Facilitator techniques (e.g. the use of flip charts, mind maps) were tabulated but this assessment was found to be problematic since we used audio- (rather than video-) recordings of courses. It was often difficult to tell which types of techniques were being used and this could not be assessed consistently. Thus this measure was omitted in the final fidelity assessment.

## Discussion

MRC guidelines state that in some cases, strict adherence to protocol can be necessary to determine the impact of each component but, in other cases, an intervention that is adaptable may allow the delivery of a programme that is better suited to the needs of a particular group of participants [3]. Increasing adherence may decrease competence, although allowing too much flexibility may undermine the delivery of core course components [8]. It thus becomes important to find how much variability is permitted before the intervention becomes compromised, and for this implementation fidelity studies are necessary [3]. The facilitators of the SMILE(UK) course were encouraged to adapt the material to match a particular group’s needs [33]. However, for our randomised controlled trial of the self-management course [5], it was important to measure the delivery of the core items thought to be important in behaviour change. In this context, we developed an instrument for intervention fidelity measuring both delivery of core items (adherence) and the way they were presented to the group (competence). Our instrument was based on previous work evaluating adherence and competence [10, 12], and included a novel measure of didacticism. Mars et al. [9] evaluated a self-management intervention for pain and measured fidelity in a similar way to our study. Their adherence check-list had an 80% inter-rater agreement, which is similar to our findings (81%). Our agreement for “overall impression” was slightly higher with 60% compared to their 53%. However, the similarities between the two studies demonstrate the robustness of this type of instrument.

To our knowledge, this is the first attempt to measure intervention fidelity for a self-management course for people with epilepsy. Current methods for measuring adherence for other types of interventions vary greatly. Some have only used facilitator self-report measures. We, in contrast, used independent raters as this reduces the risk of bias [34, 35]. Adherence has been measured for complex self-management interventions using audio-recordings of course delivery [10, 12]. This method worked well in our study when assessing the delivery of core items from a checklist. However, this worked less well when trying to assess which specific facilitator techniques were being used. For this, a visual approach would be needed, such as raters sitting in on the course or using video recordings, although both have limitations.

Our interrater reliability results indicate that our fidelity instrument was easy to use and little training of raters was needed. The novel approach to measure didacticism was highly reproducible, and offers an objective component to the fidelity assessment. While didacticism did not correlate with “overall impression” and “group interaction” for SMILE (UK), we maintain that all three components belong in an assessment of competence. Measuring competence is to evaluate abstract concepts such as flexibility, interactivity and good judgement. The “overall impression” score was used to measure this, but it is highly subjective. To make it more concrete, “group interaction” was used by specifying the number of participants needed to interact for each score. However, interaction could mean simply asking a question, not necessarily a discussion. To assess “how much participants spoke”, it was simpler to determine the proportion of facilitator speech. Our measure of competence combined didacticism with group interaction and overall impression to provide a full assessment. Taking each measure alone would not provide the whole picture of competence. For example, by solely measuring didacticism, one could think that a highly didactic session was not interactive. However, using a group interaction measure shows this may not be the case. Alternatively, if measuring group interaction alone, we would not know how the course information was conveyed (i.e., by the facilitator or by group discussion).

### Implementation fidelity of SMILE(UK)

Our method of using a checklist to measure adherence indicated that the facilitators showed a high level of fidelity to prescribed instructions. Four items were fully delivered across all sessions and all items received the maximum score in at least one of the sessions evaluated. This demonstrates that no item was so problematic that it could not be delivered. Items that were omitted were mostly related to using the workbook during the session. The idea of incorporating the workbook throughout the sessions was implemented following feedback during the pilot study. This way, the book would not feel “foreign”, but would become a familiar tool for participants during the course. Possible reasons for not having used the workbook during the course may include time constraints, not understanding the importance of the exercise, different learning abilities, or adapting to the group’s needs. While we incorporated the workbook into our adherence measurement, it is unknown whether the workbook itself has a role in behaviour change.

The SMILE(UK) course facilitators received high scores for competence. The group interaction measure showed a high level of participant involvement throughout course delivery, which was one of the main goals of the course. The sessions were rated on average as “good”, with some didactic teaching and significant participant involvement. While the percentage of didacticism varied between sessions, this did not have an impact on group interaction and was not associated with overall impression scores. This suggests that they are measuring different aspects of competence, and also reveals that highly competent facilitators can deliver what are objectively more didactic sessions whilst maintaining good group interaction. One caveat is that we evaluated only 15 recorded modules which may limit the power of the statistical analysis.

### Limitations of the study

We chose three specific modules of SMILE(UK) to evaluate for implementation fidelity. These modules were chosen as the most informative based on a pilot study. They are also largely teaching modules and thus we expected a high level of “didacticism”. Other modules which touch on personal experience with epilepsy are largely discussion-based and less structured. It would be more difficult to measure the delivery of core items in these cases, yet opening up to others about living with a condition may be related to behaviour change by increasing self-confidence [36, 37]. Ideally, implementation fidelity of all components of a complex intervention would be measured. In the context of a randomised controlled trial, evaluating the components most related to behaviour change is especially important. However, often limited resources are allocated for fidelity assessments, and rather than omitting fidelity altogether, our study, along with others [10, 12], shows that measuring the important aspects of the intervention can suffice. Had we included the less structured modules of the course, this may have had an impact on the interclass correlation coefficients. It is difficult to determine in which direction they could change as this depends on the design of the instrument. For the modules involving more discussion (e.g. Module One: Living with Epilepsy), participants are asked to talk and share their feelings about being diagnosed and living with the condition. The fidelity instrument could have been designed to assess whether every participant had spoken about these two topics, and the correlation between raters on this single question could be high. It would, however, be a disservice to facilitators to include specific topics to address, such as anger and fear, if none of the participants currently experienced these emotions.

The same can be said for the proportion of courses evaluated. Ideally the implementation fidelity of 100% of all sessions would be measured. Previous research has, however, shown that 25–40% of courses are sufficient for analysis [30]. Thus, we analysed 28% of the courses delivered. We selected courses to assess fidelity across a variety of facilitators. It is known that facilitators can differ in important ways (such as prior teaching experience and personality) and these factors may affect the way in which they deliver an intervention. On the other hand, we were not able to measure how consistent each facilitator was across the different courses they delivered or whether they improved with time. We were also unable to assess whether fidelity depended on the facilitators, or in other words, were some facilitators consistently better at delivering the course than others. How well a course is delivered, not only in terms of content provided but also how engaging the facilitators are, may impact the outcomes of the study (i.e., the enactment of learned skills by participants).

As we limited the analysis to specific modules, a low adherence score may not necessarily mean an item was not delivered during the whole course as there can be overlap between modules. Some items may have been touched on during other modules which our assessment was not able to evaluate. Our group interaction score was based on a specific number of people participating in discussion. Another way to do this, for example in a larger group setting, could to be to modify the score to reflect a certain percentage of the group, rather than using a specific number. Group size was associated with didacticism, in that facilitators spoke less in smaller groups. This emphasises that smaller groups may be a better setting for enabling people to share their experiences. For a course such as SMILE(UK), it is important to have variety in patient experience since this will enable learning between patients with diverse epilepsy histories, and, therefore it is likely that a group of a certain size is needed. The variations in our group sizes (of 6–13 participants) may have been associated with different levels of didacticism and possibly impacted on participant experiences, but this would not seem to have affected adherence or competence aspects of fidelity. We are unable to say whether larger groups (e.g. 15–20 participants) would impact fidelity.

## Conclusion

Understanding the level of adherence and competence with which an intervention was delivered is critical to determining whether it was implemented as intended. The current study offers a holistic and multi-faceted method of measuring implementation fidelity. No study to date has measured each of these dimensions in the evaluation of a complex intervention for epilepsy, and this paper presents insight into designing a reliable and valid fidelity instrument to do so. Our fidelity evaluation showed that SMILE(UK) can be implemented with high levels of adherence to core items while maintaining group interactivity.

## Additional files

Additional file 1: Raters’ checklists. Two tables are included as Additional Files. The first table is the checklist used to score adherence, group interaction and overall impression of the three modules evaluated for implementation fidelity. The second table is the checklist used to record facilitator techniques used when delivering course content. (DOCX 18 kb)

Additional file 2: Table of adherence scores per averaged per item. One table is included containing adherence scores for the different items on the checklist. (DOCX 15 kb)

## Abbreviations

EEG: Electroencephalogram
MOSES: Modular service package epilepsy
MRC: Medical research council
SMILE(UK): Self-Management for people with poorly controlled epILEpsy
UK: United Kingdom
