# Supplementary material for: Implementation fidelity of a self-management course for epilepsy: method and assessment
Source: BMC Med Res Methodol. 2017 Jul 11;17:100. doi: 10.1186/s12874-017-0373-x (PMC5504680; doi:10.1186/s12874-017-0373-x)
Supplement: Supplementary file 1 — Raters’ checklists. Two tables are included as Additional Files. The first table is the checklist used to score adherence, group interaction and overall impression of the three modules evaluated for implementation fidelity. The second table is the checklist used to record facilitator techniques used when delivering course content. (DOCX 18 kb) [file 12874_2017_373_MOESM1_ESM.docx]

**Additional file 1: Rater’s checklist**

**Table 1. Checklist used to assess adherence, group interaction and overall impression**

| **Module 3: Basic Knowledge** | **To what extent was item delivered by facilitators?** | **Rating** | **Comments** |
| --- | --- | --- | --- |
|  | 1. How do seizures develop? |  |  |
|  | 2. What are the different seizure types? |  |  |
|  | 3. What happens during a seizure? |  |  |
|  | 4. What are some examples of seizure types? |  |  |
|  | 5. Participants are facilitated to identify personal seizure type |  |  |
|  | 6. Participants facilitated to note seizure type in workbook |  |  |
|  | 7. Did facilitators engage participant involvement? |  |  |
|  | 8. Overall, was this chapter well-delivered?  (1 = poor, 2 = average, 3 = good, 4 = excellent) |  |  |
| **Module 4: Diagnosis** | 9. Things that are noticed before, during and after a seizure |  |  |
|  | 10. The importance of detailing specifics of a seizure |  |  |
|  | 11. What a doctor may need to know about a seizure |  |  |
|  | 12. Participants prompted to record details of their last seizure in workbook |  |  |
|  | 13. Importance of EEG |  |  |
|  | 14. Other diagnostic techniques (MRI, CT, etc.) |  |  |
|  | 15. Did facilitators engage participant involvement? |  |  |
|  | 16. Overall, was this chapter well-delivered?  (1 = poor, 2 = average, 3 = good, 4 = excellent) |  |  |
| **Module 6: Self-Control** | 17. Seizure triggers and how they vary |  |  |
|  | 18. Keeping a checklist of triggers |  |  |
|  | 19. Avoiding and eliminating triggers |  |  |
|  | 20. What is an aura and how might it be recognised? |  |  |
|  | 21. Countermeasures to achieve aura control |  |  |
|  | 22. Did facilitators engage participant involvement? |  |  |
|  | 23. Overall, was this chapter well-delivered?  (1 = poor, 2 = average, 3 = good, 4 = excellent) |  |  |

| **Rating Key** |  | **Did facilitators engage participant involvement?** |
| --- | --- | --- |
| 0 = Item was not delivered |  | 0 = 1 person dominating |
| 1 = Item was partially delivered |  | 1 = 2-3 interacting with facilitators |
| 2 = Item was fully delivered |  | 2 = 4+ interacting with facilitators |

**Table 2. Checklist used to assess trainer techniques**

| **Trainer techniques** | **Number of Instances** | | |
| --- | --- | --- | --- |
|  | **Module 3** | **Module 4** | **Module 6** |
| Slides |  |  |  |
| Mind maps |  |  |  |
| Videos |  |  |  |
| Dot-on-the line |  |  |  |
