# Supplementary material for: Implementation fidelity of a self-management course for epilepsy: method and assessment
Source: BMC Med Res Methodol. 2017 Jul 11;17:100. doi: 10.1186/s12874-017-0373-x (PMC5504680; doi:10.1186/s12874-017-0373-x)
Supplement: Supplementary file 2 — Table of adherence scores per averaged per item. One table is included containing adherence scores for the different items on the checklist. (DOCX 15 kb) [file 12874_2017_373_MOESM2_ESM.docx]

**Additional file 2: Adherence ratings per checklist item**

**Table 1. Adherence ratings per item**

| **Module** | **Module score**  *Mean (range)* | **Item** | **Item score**  *Mean (range)* |
| --- | --- | --- | --- |
| **Module 3: Basic Knowledge** | 1.5 (1.3 – 1.9) | How do seizures develop? | 2 (2 – 2) |
|  |  | What are the different seizure types? | 2 (2 – 2) |
|  |  | What happens during a seizure? | 1.1 (0 – 2) |
|  |  | What are some examples of seizure types? | 1.8 (1.5 – 2) |
|  |  | Participants are facilitated to identify personal seizure type | 1.7 (1 – 2) |
|  |  | Participants facilitated to note seizure type in workbook | 0.6 (0 – 2) |
| **Module 4: Diagnosis** | 1.5 (1.2 – 2) | Things that are noticed before, during and after a seizure | 2 (2 – 2) |
|  |  | The importance of detailing specifics of a seizure | 1.6 (1 – 2) |
|  |  | What a doctor may need to know about a seizure | 1.6 (1 – 2) |
|  |  | Participants prompted to record details of their last seizure in workbook | 0.9 (0 – 2) |
|  |  | Importance of electroencephalogram (EEG) | 1.8 (1 – 2) |
|  |  | Other diagnostic techniques | 1.3 (1 – 2) |
| **Module 6: Self-Control** | 1.6 (1.3 – 2) | Seizure triggers and how they vary | 2 (2 – 2) |
|  |  | Keeping a checklist of triggers | 1.5 (0.5 – 2) |
|  |  | Avoiding and eliminating triggers | 1.6 (1 – 2) |
|  |  | What is an aura and how might it be recognised? | 1.4 (0 – 2) |
|  |  | Countermeasures to achieve aura control | 1.5 (1 – 2) |
